# Transcriptomic analysis reveals upregulated host metabolisms and downregulated immune responses or cell death induced by acute African swine fever virus infection

**DOI:** 10.3389/fvets.2023.1239926

**Published:** 2023-08-31

**Authors:** Shinuo Cao, Peng Jia, Zhi Wu, Huipeng Lu, Yuting Cheng, Changchun Chen, Mo Zhou, Shanyuan Zhu

**Affiliations:** ^1^Swine Infectious Diseases Division, Jiangsu Key Laboratory for High-Tech Research and Development of Veterinary Biopharmaceuticals, Engineering Technology Research Center for Modern Animal Science and Novel Veterinary Pharmaceutic Development, Jiangsu Agri-animal Husbandry Vocational College, Taizhou, Jiangsu Province, China; ^2^Shenzhen Technology University, Shenzhen, Guangdong Province, China

**Keywords:** African swine fever virus, transcriptome, metabolism, innate immune response, cell death

## Abstract

The African swine fever virus is a virulent and communicable viral disease that can be transmitted by infected swine, contaminated pork products, or soft tick vectors. Nonstructural proteins encoded by ASFV regulate viral replication, transcription, and evasion. However, the mechanisms underlying the host response to ASFV infection remain incompletely understood. In order to enhance comprehension of the biology and molecular mechanisms at distinct time intervals (6, 12, 24 h) post infection, transcriptome analyses were executed to discern differentially expressed genes (DEGs) between ASFV and mock-infected PAMs. The transcriptomic analysis unveiled a total of 1,677, 2,122, and 2,945 upregulated DEGs and 933, 1,148, and 1,422 downregulated DEGs in ASFV- and mock-infected groups at 6, 12, and 24 h.p.i.. The results of the transcriptomic analysis demonstrated that the infection of ASFV significantly stimulated host metabolism pathways while concurrently inhibiting the expression of various immune responses and cell death pathways. Our study offers crucial mechanistic insights into the comprehension of ASFV viral pathogenesis and the multifaceted host immune responses. The genes that were dysregulated may serve as potential candidates for further exploration of anti-ASFV strategies.

## Introduction

African swine fever (ASF) is a viral disease of swine caused by the African swine fever virus (ASFV) (1). The disease is highly prevalent in eastern and southern Africa, where the ASFV persists through a sylvatic cycle involving warthogs (*Phacochoerus aethiopicus*) or bushpigs (*Potamochoerus* spp.) and soft tick vectors of the *Ornithodoros moubata*. It can also occur through a domestic cycle that includes indigenous breeds of pigs, with or without ticks (2). Additionally, ASFV has spread persistently throughout the *Iberian Peninsula* due to the presence of the soft tick vector *O. erraticus*. ASFV belongs to the Asfaviridae virus family and is responsible for inducing acute hemorrhagic fever in both domestic and wild pigs (3). Acute ASF infection leads to a clinical course resembling viral hemorrhagic fever, resulting in 100% mortality (4). In the absence of vaccines against ASF, its control becomes even more challenging. Currently, it is one of the largest threats to the worldwide swine industry, causing global trade to suffer (4).

The host has developed a comprehensive and interconnected defense network comprising innate and adaptive immune responses to combat viral infections. Recent studies have unveiled that several protective mechanisms, aiming to impede viral replication, dissemination, or persistent infection, involve the activation of programmed cell death (5–7). This biological process encompasses various forms of cell death, including apoptosis, autophagy, necrosis, and atypical cell death (e.g., anoikis, paraptosis, pyroptosis, and ferroptosis), playing a crucial role in physiological growth and development (8, 9). Pyroptosis, distinct from other forms of cell death, is associated with inflammation and holds significant importance in the host’s defense against infections. It achieves this through the release of proinflammatory cytokines and cell lysis (10). Conversely, autophagy is a lysosome-dependent catabolic process characterized by the formation of double-membrane autophagosomes. These autophagosomes sequester cytoplasmic components for subsequent degradation upon fusion with lysosomes. Autophagy functions as a protective mechanism for breaking down surplus or impaired cellular components, although excessive autophagic activity may lead to cell death (11). Ferroptosis, an iron-dependent form of programmed cell death, has been associated with various pathologies related to tissue injury, contributing to cellular depletion through ferroptotic cell death.

Inflammasomes and pattern recognition receptors (PRRs) are crucial components of the innate immune system’s response to viral infections. These receptors recognize conserved viral pathogen-associated molecular patterns (PAMPs), initiating downstream signaling pathways that trigger the production of type I interferon (IFN). Binding of IFNs to the IFNAR receptor on the cell membrane activates the JAK–STAT signaling pathway, which, in turn, regulates the expression of numerous interferon-stimulated genes (ISGs). Together, these ISGs establish an antiviral state that effectively combats viral infections. However, studies on investigating the response of swine macrophages to interferon-gamma (IFN-γ) and lipopolysaccharide (LPS) during ASFV infection have shown suppressed levels of interleukin-1 (IL-1) and tumor necrosis factor (TNF), indicating a modulation of the immune response (12, 13). Inflammasomes, innate immune mechanisms that activate caspase-1 protease, promote inflammation, have been found to induce both pyroptosis and apoptosis based on recent research findings. Conversely, the regulation of transcriptional co-activator proteins p300 and cyclic AMP response element-binding protein (CBP) by ASFV appears to modulate the expression of TNF-α and iNOS (14–16). However, conflicting evidence regarding the role of inflammatory mediators in ASFV infection has been reported by various studies.

Currently, the mechanisms by which ASFV modulates immune response and cell death pathways remain incompletely elucidated (17). Therefore, it is crucial to comprehensively assess the immune cell response during ASFV infection. In this study, we performed a temporal analysis of gene expression dynamics, including both host and viral genes, and conducted enriched functional annotations. Our findings demonstrate that the differentially expressed genes (DEGs) are mainly involved in metabolic processes, immune responses, and cell death. Transcriptomic analysis holds the potential to enhance our understanding of host immune responses and cell death pathways in the context of ASFV infection, providing valuable insights for the development of antiviral strategies and resistance against viral escape.

## Materials and methods

### Data preprocessing and analysis of differential gene expression

In this study, the transcriptomics dataset GSE145954 of ASFV was utilized. To preprocess the data, genes with low expression (i.e., sum of gene counts for all samples <100) were removed from the dataset (18). Subsequently, differential gene expression analysis was conducted using the limma package in the R language (19, 20). The identification of genes that were expressed differentially was carried out using Benjamini-Hochberg FDR correction techniques with adjusted *p*-values (FDR < 0.05) and statistical threshold parameters of |log2(FC)| ≥ 1.

### Gene ontology and pathway enrichment analysis

The present study utilized DAVID and KOBAS online analyses to perform gene ontology (GO) and Kyoto Encyclopedia of Genes and Genomes (KEGG) pathway enrichments of DEGs. Specifically, the DAVID database was employed as a fundamental tool for high-throughput gene function analysis, and was utilized to analyze the functional and pathway enrichment of the DEGs. Additionally, KOBAS online analysis database was utilized to perform GO enrichment and KEGG pathway analysis of the DEGs. The present investigation involved an analysis of the DEGs that exhibited significant up- and down-regulation in porcine alveolar macrophages infected with ASFV. To identify noteworthy GO terms and KEGG pathways, a statistical threshold criterion was applied, wherein an adjusted *p*-value of less than 0.05 was utilized.

### Expression profiling of apoptosis, autophagy, and ferroptosis-related genes

To visualize the DEGs, a volcano plot was utilized. The pyroptosis-related genes (PRGs) were extracted from the MsigDB,^1^ while Human Autophagy Database (HADb, http://www.autophagy.lu/) was used to obtain autophagy-related genes (ARGs). From the FerrDB,^2^ 144 validated iron death-associated genes were retrieved. The identification of autophagy-related genes that were differentially expressed was accomplished by selecting the intersection of the DEGs and ARGs, which were subsequently evaluated through the use of a heatmap.

### Expression profiling of cytokines and inflammatory response, cytosolic DNA sensing pathway, and interferon-stimulating genes

The immune response in PAMs infected with ASFV was subsequently investigated. Heatmaps were constructed using a compilation of genes from KEGG_CHEMOKINE_SIGNALING_PATHWAY (M4844) cytokines and inflammatory response, as well as BIOCARTA_INFLAM_PATHWAY (M6910) cytosolic DNA sensing pathway. The former consisted of 217 genes, while the latter comprised 56 genes. To examine ISG expression, a comprehensive list of 644 ISGs was generated from published literature (7, 21).

### Construction of PPI network and community discovery analysis

The protein–protein interactions (PPI) of DEGs were obtained from the STRING database through the utilization of the R package STRINGdb, with default parameters (version = “11,” species = 9,606, score_threshold = 400, network_type = “full”). The Cytoscape software (version 3.9.1, http://chianti.ucsd.edu/cytoscape-3.9.1/) was utilized to visualize the PPI network based on the interactions obtained. In addition, the Molecular Complex Detection (MCODE) plugin from Cytoscape software was applied to identify core gene modules with scores greater than 10 (Degree cutoff = 2; Node score cutoff = 0.2; K-core = 2 and Max depth = 100).

### Identification of hub genes

The identification of hub genes within a gene module, characterized by a high degree of connectivity, serves to investigate the properties of a module. Furthermore, the determination of a module’s connectivity, which defines the hub genes, was accomplished through the utilization of the absolute value of Pearson’s correlation (|cor.geneModuleMembership| > 0.8) (22). The genes of the key modules were uploaded into the STRING database, with a confidence score cutoff of >900 selected for the construction of protein–protein interactions (PPI). Hub genes were defined as genes with a connectivity degree of ≥8 in the protein–protein interaction (PPI) network (23).

### Statistical analysis

The statistical significance of the results was assessed through the utilization of GraphPad Prism 8 (San Diego, CA, United States). The data are presented as the mean values ± standard errors (SEs) derived from three independent experiments. A significance level of *p < 0.05 was deemed statistically significant, while **p < 0.01 and ***p < 0.001 were considered highly statistically significant.

## Results

### Transcriptional landscapes reveal ASFV-driven distinct host gene transcription at 6, 12 and 24 h.p.i.

**Figure 1:**
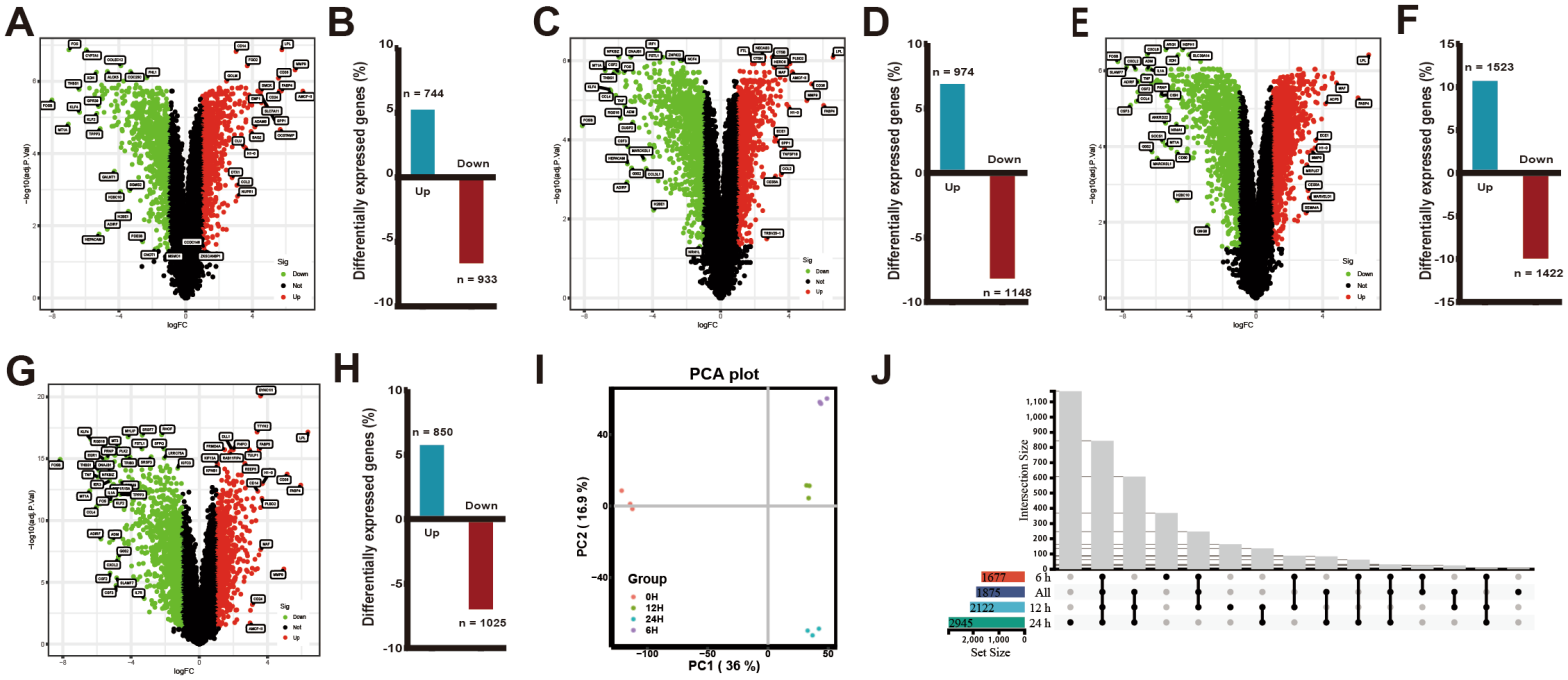
Changes in differential gene expression in PAMs at different times after ASFV infection. **(A,C,E,G)** Volcano plots showing fold changes and adjusted *p*-values for genes differentially expressed between unstimulated (mock) and ASFV-stimulated PAMs at 6, 12, and 24 h.p.i., respectively. **(B,D,F,H)** The upregulated/downregulated and the total number of DEGs (≥2 FC, *p* < 0.05) between unstimulated (mock) and ASFV-stimulated PAMs in the transcriptomic data at 6, 12, and 24 h, respectively. **(I)** The principal component analysis (PCA). **(J)** Venn diagrams show an overlap of ASFV-induced DEGs across 6, 12, 24 h.p.i. and the integrated data of the three time points.

Genes exhibiting a fold change of at least two-fold (FC) at 6, 12, and 24 h post-infection (h.p.i.) were designated as DEGs with a significance level of *p* < 0.05 (Figures 1A–F). Principal component analysis (PCA) was employed to identify dissimilarities among the various samples.

The extent of DEGs during ASFV infection was determined by comparing the transcriptome profiles of the ASFV-infected and mock-infected groups at various time points (Figure 1I). The results depicted in Figures 1A–F indicated that the number of DEGs (*p*-value <0.05 and |log2FC| > 1) was 1,677, 2,122, and 2,945 at 6, 12, and 24 h.p.i., respectively, with a majority of the DEGs being upregulated or downregulated at 24 h.p.i.. Moreover, at 6, 12, and 24 h.p.i., 744, 974, and 1,523 genes were upregulated, respectively, while 933, 1,148, and 1,422 genes were downregulated. In total, 1875 DEGs were identified following ASFV infection, with 850 (45%) genes exhibiting upregulation and 1,025 (55%) genes exhibiting downregulation in the integrated transcriptome data analysis across the three time points (Figures 1G–H). The Venn diagram illustrated the genes that were either exclusive or common at each time point (Figure 1J). The findings revealed that 844 DEGs were shared among four gene sets, while the unique DEGs for each gene set were 369, 8, 164, and 1,170, respectively.

### ASFV-infection promoted metabolic pathways and suppressed the immune response of host for transcriptomic analysis of DEGs

**Figure 2:**
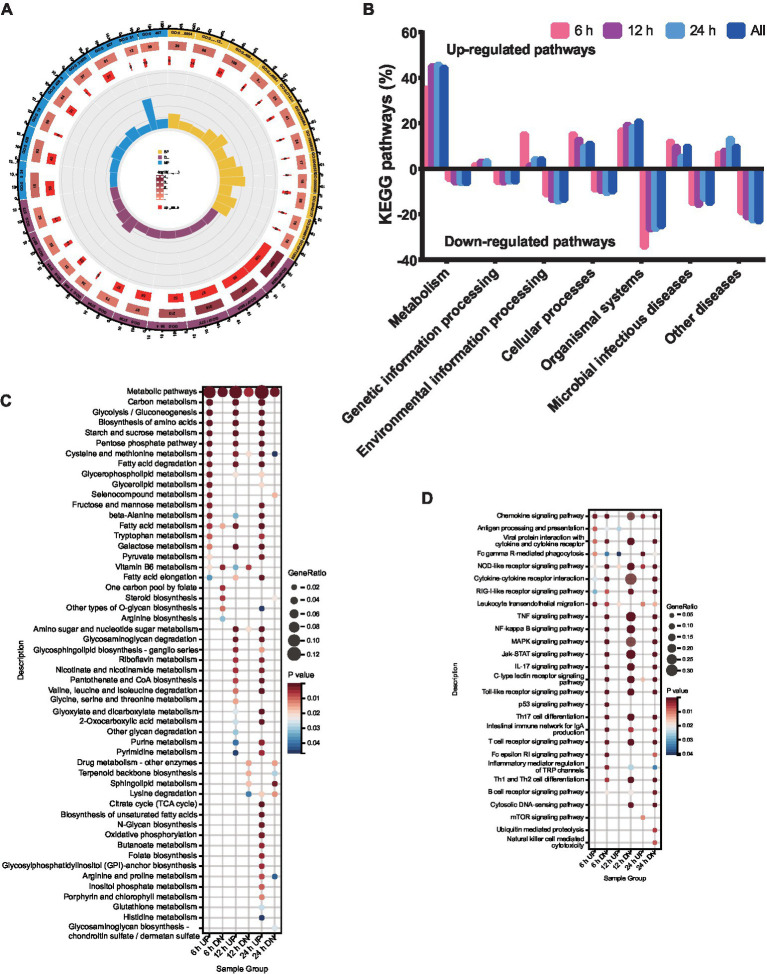
Functional enrichment analysis of DEPs. DEPs were classified according to their biological process, cellular component, molecular function, domain, and KEGG based on GO and KEGG enrichment analysis. **(A)** GO analysis of the genes with expression changes for the integrated data of the three time points. **(B)** KEGG analysis of the genes with expression changes at 6, 12, 24 h.p.i. and the integrated data. **(C)** KEGG enrichment analysis of metabolic pathways. **(D)** KEGG enrichment analysis of immunity pathways.

In order to discern the biological process patterns impacted by ASFV infection, the DEGs were subjected to GO enrichment mapping. The DEGs that emerged subsequent to ASFV infection were predominantly implicated in biological processes associated with the inflammatory response, immune response, negative regulation of apoptotic process, positive regulation of tyrosine phosphorylation of STAT protein, mitotic cytokinesis, regulation of cell population proliferation, and activation of cysteine-type endopeptidase activity involved in apoptotic process. The molecular function category exhibited enrichment of ATP binding, identical protein binding, protein kinase binding, protein homodimerization activity, ubiquitin protein ligase binding, zinc ion binding, mitogen-activated protein kinase binding, and protein serine/threonine kinase activity, as depicted in Figure 2A.

To explore the functional pathways of DEGs in porcine alveolar macrophages infected with ASFV, KEGG analysis was performed on the DEGs (Figure 2B). The KEGG enrichment analysis revealed a significant upregulation of metabolism pathways, while the environmental information processing pathways were significantly downregulated at 12 and 24 h.p.i., with the exception of 6 h.p.i.. The genetic information processing was significantly downregulated at all time points. Notably, the number of pathways involved in host metabolism exhibited an increase from 6 to 24 h.p.i., as indicated by the KEGG analysis (Figure 2C). Furthermore, the pathways related to immune response were significantly downregulated at 6, 12, and 24 h.p.i. (Figure 2D).

### Transcriptomic profiles of apoptosis, autophagy, and ferroptosis-related genes responding to ASFV infection

**Figure 3:**
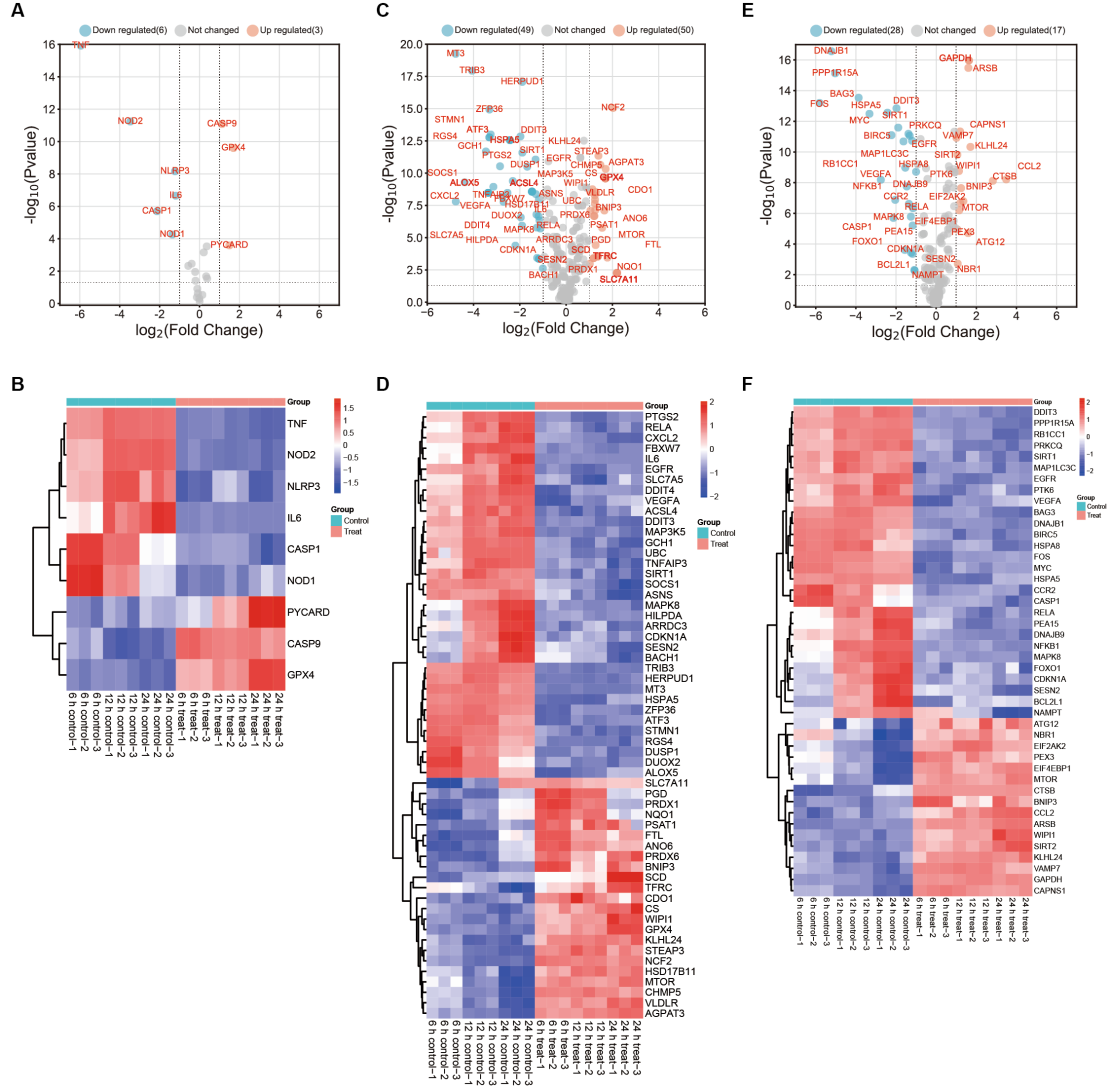
DEGs in PAMs after *in vitro* ASFV-specific stimulation reveal concomitant apoptosis, autophagy, and ferroptosis responses. **(A,C,E)** Volcano plots showing fold changes and adjusted *p*-values for ASFV DEGs for apoptosis, autophagy, and ferroptosis between unstimulated (mock) and ASFV-stimulated cells from unvaccinated and vaccinated pigs. **(B,D,F)** Heatmap analysis of ASFV DEGs for apoptosis, autophagy and ferroptosis at different time points post-ASFV infection.

The present study utilized a volcanic map and heatmap to obtain the expression profile matrix of genes related to apoptosis. The findings indicate that significant expression differences exist between ASFV-infected and mock-infected samples with respect to apoptosis-related genes. Specifically, six apoptotic genes (TNF, NOD2, NLRP3, IL6, CASP1, and NOD1) in the apoptosis-activating pathway were found to be significantly downregulated in ASFV-infected PAMs, whereas three apoptotic genes (PYCARD, CASP9, GPX4) were upregulated (Figures 3A,B).

The results showed that a significant decrease in the expression of thirty-five autophagy-associated genes, including PTGS2, RELA, CXCL2, FBXW7, IL6, EGFR, SLC7A5, DDIT4, VEGFA, ACSL4, DDIT3, MAP3K5, GCH1, UBC, TNFAIP3, SIRT1, SOCS1, ASNS, MAPK8, HILPDA, ARRDC3, CDKN1A, SESN2, BACH1, TRIB3, HERPUD1, MT3, HSPA5, ZFP36, ATF3, STMN1, RGS4, DUSP1, DUOX2, and ALOX5, in cells infected with ASFV compared to mock-infected cells (Figures 3C,D). The study observed a significant upregulation in the expression of twenty-three autophagy-associated genes, including SLC7A11, PGD, PRDX1, NQO1, PSAT1, FTL, ANO6, PRDX6, BNIP3, SCD, TFRC, CDO1, CS, WIPI1, GPX4, KLHL24, STEAP3, NCF2, HSD17B11, MTOR, CHMP5, VLDLR, and AGPAT3, in ASFV-infected cells compared to mock-infected cells. Additionally, CXCL2 was identified as one of the top 10 most-induced genes after ASFV infection, exhibiting a > 8-fold downregulation.

The ASFV infection leads to a significant downregulation of twenty-eight ferroptosis-associated genes, including DDIT3, PPP1R15A, RB1CC1, PRKCQ, SIRT1, MAP1LC3C, EGFR, PTK6, VEGFA, BAG3, DNAJB1, BIRC5, HSPA8, FOS, MYC, HSPA5, CCR2, CASP1, RELA, PEA15, DNAJB9, NFKB1, MAPK8, FOXO1, CDKN1A, SESN2, BCL2L1, and NAMPT, when compared to mock-infected cells. Conversely, sixteen ferroptosis-associated genes, namely ATG12, NBR1, EIF2AK2, PEX3, EIF4EBP1, MTOR, CTSB, BNIP3, CCL2, ARSB, WIPI1, SIRT2, KLHL24, VAMP7, GAPDH, and CAPNS1, were found to be significantly upregulated in ASFV-infected cells as compared to mock-infected cells (Figures 3E,F).

### ASFV infection triggers host innate immune responses

**Figure 4:**
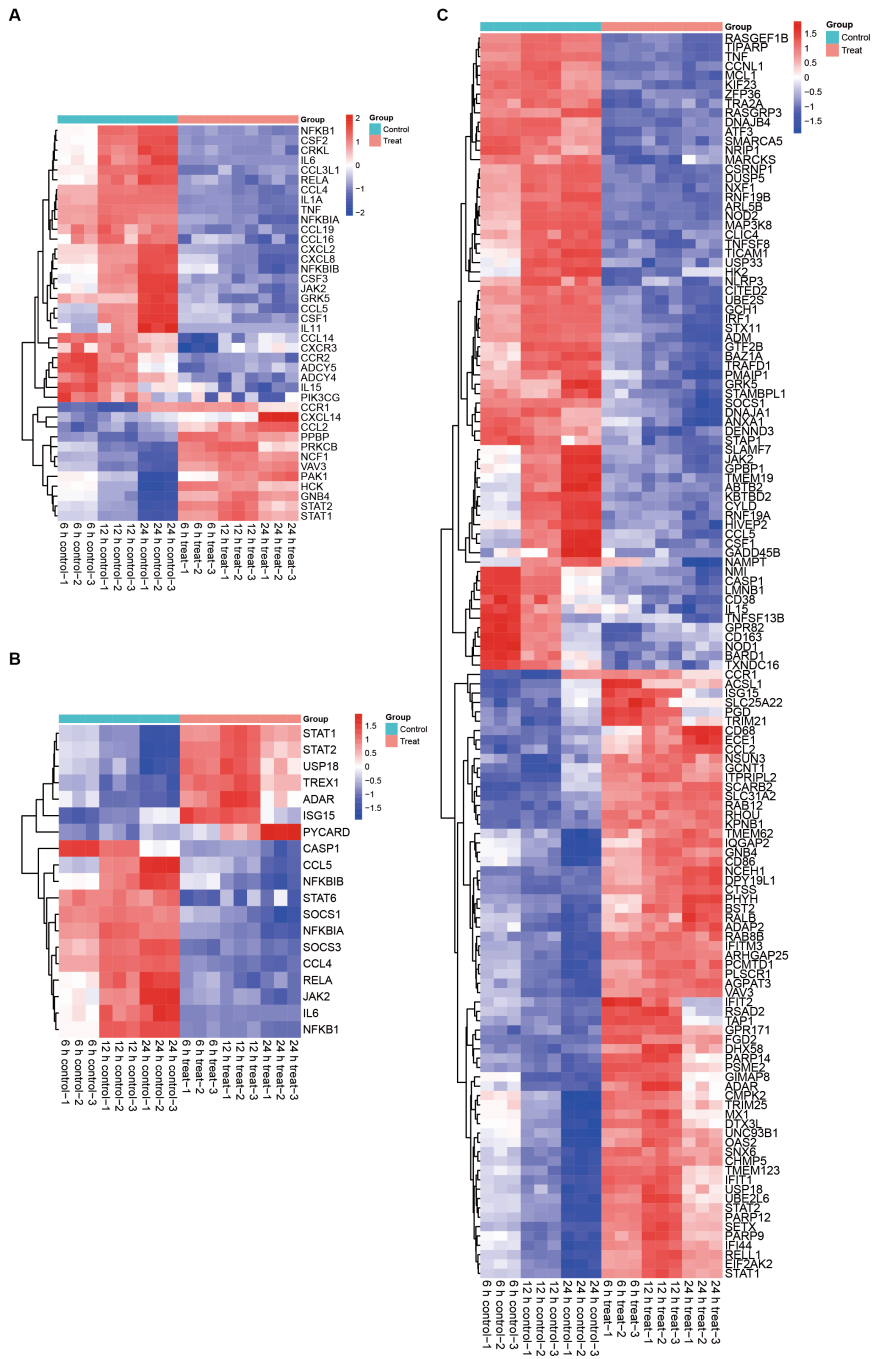
Effect of ASFV infection on the transcription of anti-viral factors, cytokines, chemokines, infammatory, JAK–STAT associated genes and ISGs in ASFV-infected PAMs. **(A)** Heatmap showing expression profiles of 123 DEGs encoding ISGs. **(B)** Heatmap of 46 DEPs associated with ISGs. **(C)** Heatmap showing expression profiles of 70 DEGs encoding chemokine signaling pathway, and cytokine-cytokine receptor interaction related proteins.

The expression of twelve chemokines, cytokines, and inflammatory response markers (CCR1, CXCL14, CCL2, PPBP, PRKCB, NCF1, VAV3, PAK1, HCK, GNB4, STAT2, and STAT1) was significantly induced by ASFV infection, while the expression of NFKB1, CSF2, CRKL, IL6, CCL3L1, RELA, CCL4, IL1A, TNF, NFKBIA, CCL19, CCL16, CXCL2, CXCL8, NFKBIB, CSF3, JAK2, GRK5, CCL5, CSF1, IL11, CCL14, CXCR3, CCR2, ADCY5, ADCY4, IL15, and PIK3CG was suppressed (Figure 4A).

Based on the quantifiable transcriptomic data set, it was determined that the expression levels of STATs were increased subsequent to ASFV infection. Specifically, the seven JAK–STAT associated genes, namely STAT1, STAT2, USP18, TREX1, ADAR, ISG15, and PYCARD, exhibited significant upregulation in ASFV-infected cells compared to mock-infected cells. Conversely, the expression of twenty-eight ferroptosis-associated genes, including CASP1, CCL5, NFKBIB, STAT6, SOCS1, NFKBIA, SOCS3, CCL4, RELA, JAK2, IL6, and NFKB1, was significantly decreased in ASFV-infected cells relative to mock-infected cells (Figure 4B).

The involvement of STATs as crucial molecules in the JAK–STAT signaling pathway and their modification’s essentiality for the production of ISGs is well-established. Our transcriptomic analysis provides further evidence for this proposition, as we observed a significant upregulation of sixty-five ISGs following ASFV infection. Conversely, we noted a significant downregulation of sixty-seven JAK–STAT downstream genes in ASFV-infected cells compared to mock-infected cells (Figure 4C). Collectively, these findings indicate that ASFV infection activates the JAK–STAT pathway and stimulates ISGs production.

### Construction and subcluster analysis of PPI network

**Figure 5:**
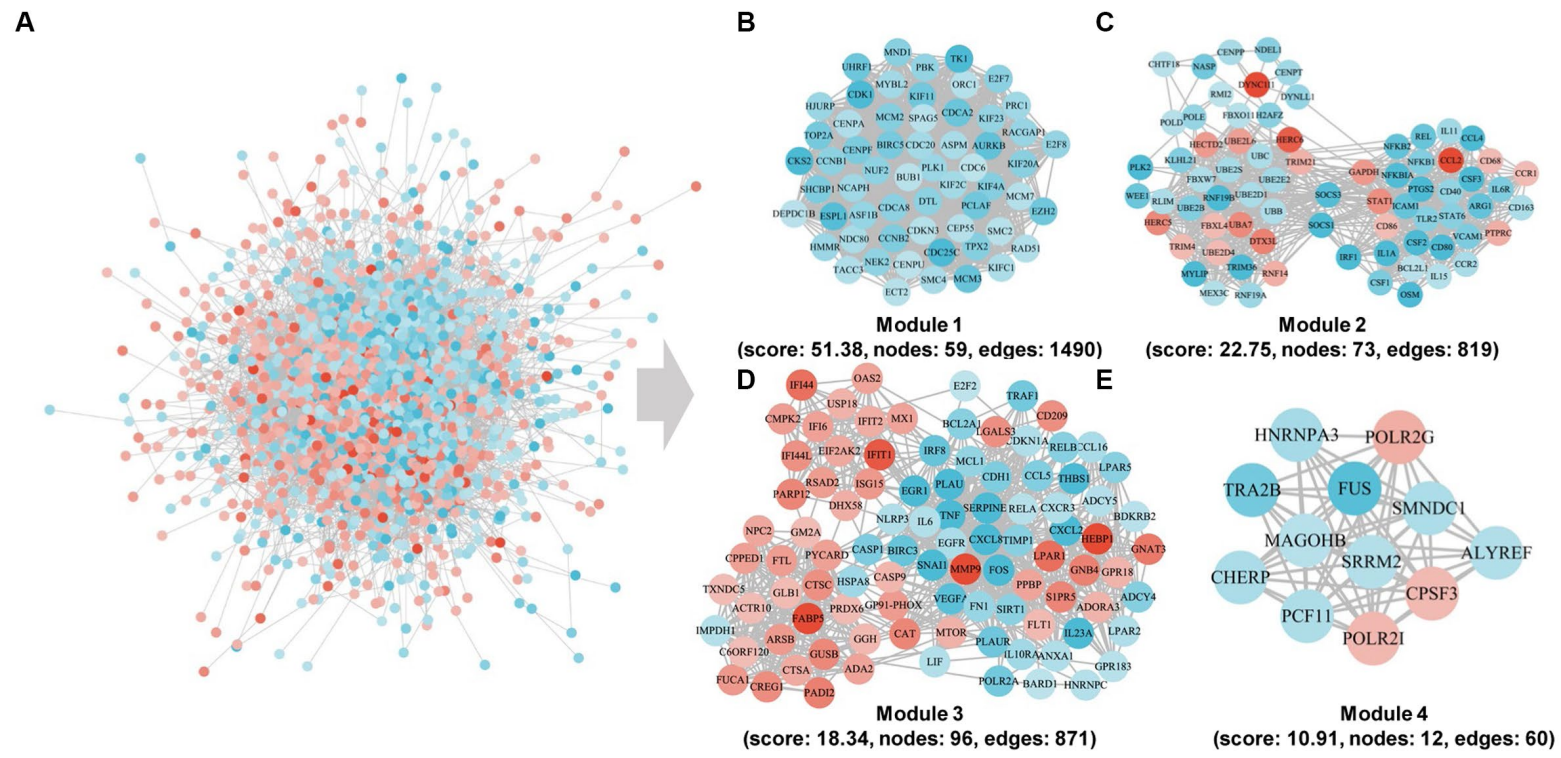
Community discovery clustering network of DEGs in the integrated data of the three time points. **(A)** PPI subnetwork. **(B–E)** PPI network of cluster1, cluster2, cluster3, and cluster4 genes.

Subsequent to our initial investigation, we delved deeper into the interactions among the DEGs and proceeded to construct a protein–protein interaction (PPI) network, as depicted in Figure 5. From this network, we identified four distinct modules and conducted an enrichment analysis of the genes within each module. Notably, the 59 genes comprising module 1 exhibited significant enrichment in pathways related to progesterone-mediated oocyte maturation, oocyte meiosis, cell cycle, and DNA replication. Module 2 comprises 73 genes that have been associated with various pathways, such as Ubiquitin mediated proteolysis, TNF signaling pathway, Rheumatoid arthritis, JAK–STAT signaling pathway, Cytokine-cytokine receptor interaction, and NF-kappa B signaling pathway. In contrast, Module 3 comprises 96 genes that exhibit enrichment in defense response to virus, cellular response to lipopolysaccharide, inflammatory response, negative regulation of viral genome replication, innate immune response, and immune response, thereby indicating their potential involvement in immune-related processes. Lastly, the 12 genes in module 4 are enriched in spliceosome, mRNA surveillance pathway, RNA polymerase, and amyotrophic lateral sclerosis.

### Screening of molecular markers associated with ASFV immunopathology based on machine learning methods

**Figure 6:**
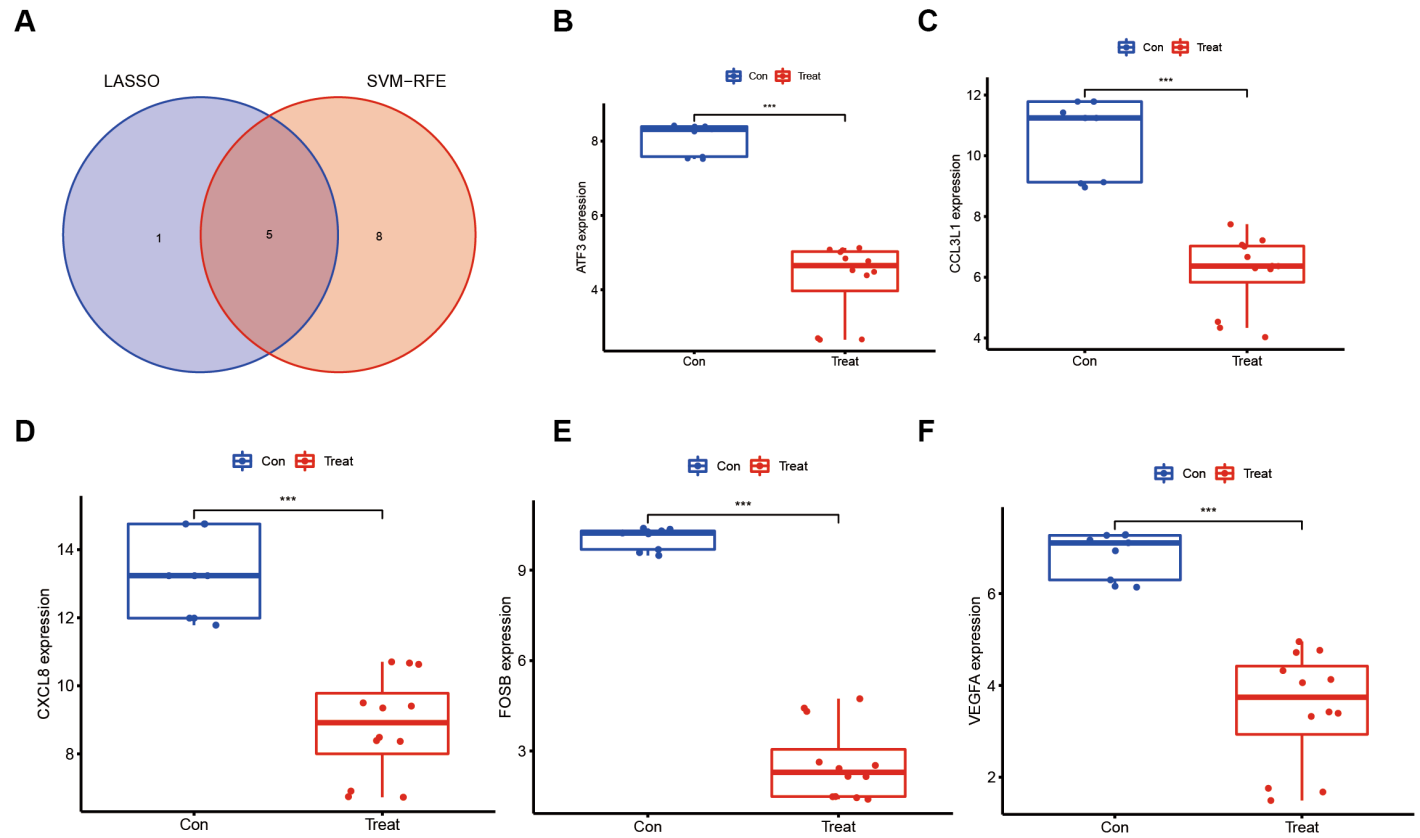
Molecular markers associated with ASFV immunopathology based on machine learning methods. **(A)** Venn diagrams show an overlap of molecular markers associated with ASFV immunopathology based on support vector machine (SVM) and least absolute shrinkage and selection operator (LASSO). **(B–F)** The molecular markers including ATF3, CCL3L1, CXCL8, FOSB, and VEGFA were significantly downregulated in ASFV-infected cells than in mock-infected cells.

The utilization of machine learning techniques to identify biomarkers for ASFV infection offers a fundamental framework for investigating the molecular mechanisms underlying ASFV infection, as well as for diagnosing and treating the infection at the genetic level. To reduce the data dimensions, a software defect prediction (SDP) model was employed, which relied on support vector machine (SVM) and least absolute shrinkage and selection operator (LASSO) (Figure 6A). Ultimately, the five genes, namely ATF3, CCL3L1, CXCL8, FOSB, and VEGFA, were identified as feature genes. Based on the quantifiable transcriptomic data set, it was observed that the expression levels of these five feature genes were significantly lower in ASFV-infected cells as compared to mock-infected cells (Figures 6B–F).

## Discussion

African Swine Fever is distinguished by the presence of haemorrhages in lymphoid organs and severe lymphoid depletion in clinical settings (24). The monocytes-macrophages are the primary target cells of ASFV (25). Investigating the immune responses in ASFV-infected PAMs can aid in elucidating the pathogenetic mechanisms underlying ASF. High-throughput technologies that detect mRNA expression levels can facilitate an integrative analysis of transcriptomic data, enabling the measurement of global changes of proteins in host cells infected with RNA- and DNA viruses (26, 27). ASFV strains exhibit varying levels of virulence towards the same host. The HLJ/18 strain, for instance, is highly virulent and easily transmissible among pigs, but the underlying mechanisms of infection and pathogenesis remain incompletely understood (28). To address this knowledge gap, we conducted transcriptomic analyses to comprehensively investigate the innate immune responses of ASFV HLJ/18-infected PAMs. One key advantage of integrative transcriptomic analyses is their ability to yield diverse results due to post-transcriptional machinery and post-translational modifications. Such studies may ultimately provide valuable insights into the pathogenicity of ASFV and its impact on host immune responses.

The mechanisms of pathogenicity and the regulation of host immune response by the ASFV are crucial in the development of vaccines. The utilization of transcriptomic analysis proves to be a proficient approach in tackling various clinical inquiries, as evidenced by the identification of distinct variances in gene expression and biological processes. The antiviral properties of PAMs are crucial in safeguarding the swine lung. Polarized macrophages exhibit differentiation into two distinct states, the pro-inflammatory M1 and the anti-inflammatory M2, based on their activation (29, 30). ASFV, being a DNA virus, effectively disrupts host immune responses and manipulates immune cell mechanisms, thereby antagonizing host defense (25, 26). In order to evade detection by the immune system, the virus has the ability to modulate the expression of pro-inflammatory molecules and cytokines of the host cells. However, the precise mechanism by which the ASFV alters the phenotype of the host immune cell and the host recognition process remain unclear. Transcriptome analysis of ASFV Georgia 2007 strain infected cells revealed up-regulation of twenty cytokines, including IFN-α, IFN-β, IFN-γ, IL-1β, and TNF superfamily cytokines, and down-regulation of four cytokines, including IL-10, IL-16, TNFSF11, and THFSF15 (31, 32). Our results indicated that ASFV-infection promotes numerous metabolic pathways and suppresses the immune response of host. Furthermore, a recently published study characterized transcriptomes of porcine macrophages afflicted with ASFV using single-cell RNA sequencing and unveiled the induction of antiviral signaling pathways, alongside heightened expression levels of interferon-stimulated genes and genes associated with inflammation and cytokine responses (33).

Moreover, antecedent literature has demonstrated that infection with ASFV of varying virulence can give rise to disparate inflammatory reactions, immune responses, and apoptotic pathways, while the pertinent mechanisms remain enigmatic. The transcriptome analysis reveals that ASFV infection leads to a significant regulation of multiple ISGs and cytokines. The expression of ISGs is strongly induced by ASFV, which may contribute to the immune pathogenicity of the virus (16, 26). The host’s multifaceted antiviral response is largely mediated by type I IFNs (IFN-α and IFN-β), which play a critical role in inducing the expression of ISGs. The JAK–STAT pathway is a crucial signaling pathway that is activated downstream of IFN, leading to the production of ISGs and the establishment of an antiviral state during viral infection. However, the mechanisms by which ASFV modulates this pathway have been sparsely reported. Our transcriptomics analyses have demonstrated that the apoptosis, autophagy, and ferroptosis-related genes in PAMs are significantly regulated after ASFV infection. In conjunction with prior investigations, we have identified and analyzed numerous regulatory pathways and compelling targets of action (32–35). These revelatory outcomes are poised to furnish profound insights for subsequent explorations aimed at offering valid data for screening potential targets for ASFV inhibition and comprehending the host response subsequent to ASFV infection.

## Conclusion

In summary, we conducted a comparative and analytical examination of the DEGs in ASFV-infected PAMs at distinct time intervals (6, 12, 24 h.p.i.). The study revealed a noteworthy increase and alteration of transcriptomic factors across various pathways, including metabolism, genetic information processing, environmental information processing, cellular processes, organismal systems and microbial infectious diseases. Transcriptomic analysis indicated that acute ASFV infection promoted host metabolisms and suppressed immune responses and cell death. The present study contributes novel insights into the immunopathogenesis of ASFV infection, which can aid in the development of vaccines and small molecule compounds to mitigate the threat of ASF, as well as in the exploration of the pathogenic mechanisms underlying this disease.

## Data availability statement

The datasets presented in this study can be found in online repositories. The names of the repository/repositories and accession number(s) can be found in the article/supplementary material.

## Author contributions

The study was conceived and designed by MZ, SZ, and PJ, while SC and PJ authored the manuscript. SZ and PJ provided critical revisions to the manuscript. The majority of the experiments were conducted by SC, PJ, ZW, HL, YC, CC, and MZ. All authors contributed to the article and approved the submitted version.

## Funding

This study received financial support from various sources, including a grant from the key project of Jiangsu Province’s Key Research and Development plan (modern Agriculture) (BE2020407), the funding of Swine Infectious Diseases Division (NSF2023TC01), the project of Jiangsu Agri-animal Husbandry Vocational College (NSF2022CB04, NSF2022CB25), the Natural Science Research Project of Higher Education of Jiangsu Province (2020220375), the Qing Lan Project of Jiangsu Province, and the 2020 Taizhou Science and Technology Support Plan (Agriculture) Project (TN202001).

## Conflict of interest

The authors declare that the research was conducted in the absence of any commercial or financial relationships that could be construed as a potential conflict of interest.

## Publisher’s note

All claims expressed in this article are solely those of the authors and do not necessarily represent those of their affiliated organizations, or those of the publisher, the editors and the reviewers. Any product that may be evaluated in this article, or claim that may be made by its manufacturer, is not guaranteed or endorsed by the publisher.

## Footnotes

^1^ http://www.gsea-msigdb.org/gsea/msigdb/index.jsp

^2^ http://www.zhounan.org/ferrdb
